# The tail of chlorophyll: Fates for phytol

**DOI:** 10.1016/j.jbc.2021.100802

**Published:** 2021-05-19

**Authors:** Timothy P. Durrett, Ruth Welti

**Affiliations:** 1 Department of Biochemistry and Molecular Biophysics, Kansas State University, Manhattan, Kansas, USA; 2 Division of Biology, Kansas State University, Manhattan, Kansas, USA

**Keywords:** catabolism, chlorophyll, chlorophyll metabolism, chlorotic plant stress, phytenal, phytol, photosynthesis, CoA, Coenzyme A

## Abstract

Understanding the pathways involved in chlorophyll breakdown provides a molecular map to the color changes observed in plant life on a global scale each fall. Surprisingly, little is known about the fate of phytol, chlorophyll’s 20-carbon branched-chain tail, during this process. A recent study from Gutbrod *et al.* provides evidence using physiological, genetic, and exquisitely sensitive analytical approaches that phytenal is an intermediate in plant phytol catabolism. These insights and techniques open the door to further investigation of this complicated metabolic system, with implications for plant health and agriculture.

Most life on earth depends on photosynthesis, which depends on the pigment chlorophyll. Chlorophyll turns over during the life of plants, and degradation is particularly rapid when leaves senesce or when plants experience specific stresses, such as salt exposure, nitrogen deprivation, or extended darkness, that induce chlorosis. Chlorophyll breakdown enables recycling of its components, including its phytyl side chain. However, the turnover pathway of the phytyl side chain has been difficult to determine as one suspected intermediate is both toxic to cells, limiting its accumulation by artificial means, and prone to modification during extraction. In their study, Gutbrod *et al.* (1) develop and use sensitive analytical methods to identify the suspected compound as an intermediate in chlorophyll degradation, establishing a key step in the degradative pathway for the phytyl chain of chlorophyll in plants.

Chlorophyll *a*, the most common plant chlorophyll, is composed of a chlorin ring (chlorophyllide), formed *via* the porphyrin biosynthetic pathway, and a phytyl side chain, an isoprenoid alcohol synthesized by reduction of a geranylgeranyl group, after its transfer to chlorophyllide from geranylgeranyl diphosphate (Fig. 1) (2). The chlorin ring binds a magnesium ion in its center and is anchored to the thylakoid membrane by the phytyl chain, which, with 20 carbons, represents over a third of the mass of chlorophyll *a*. When chlorophyll *a* degrades, it loses its magnesium, forming pheophytin, which then can be hydrolyzed by pheophytinase to form the alcohol phytol and pheophorbide *a*. While the breakdown of pheophorbide *a* has been well characterized (3), the fates of phytol have been explored only more recently. In plants, phytol can be used to synthesize tocopherols (vitamin E; (4,5)), which serve as antioxidants and potentially as signaling compounds, to make phylloquinone (vitamin K1; (6)), an electron carrier in photosystem I, or temporarily sequestered as esters with fatty acids (7), possibly to enable reuse when stress conditions have passed. In mammals, phytol is degraded by conversion to phytenal, phytenic acid, phytenoyl–coenzyme A (CoA), and phytanoyl–CoA (8), before degradation by α- and β-oxidation. However, in plants, the evidence for a corresponding degradative pathway has been largely lacking, except for identification of phytanoyl–CoA, which accumulates during dark stress (9) and the tentative identification of phytenal in senescing oat leaves (10).

**Figure 1:**
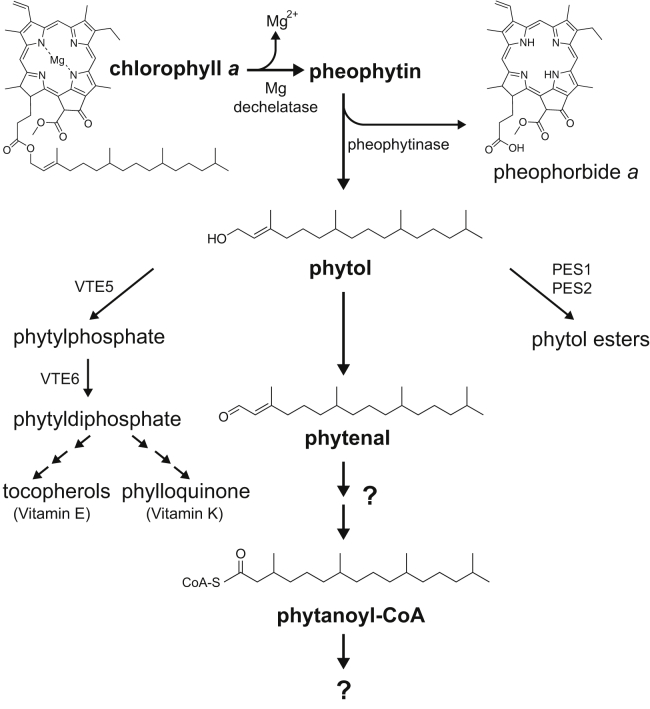
**Multiple fates for phytol after removal from chlorophyll.** During chlorophyll degradation, phytol is cleaved from the chlorin ring. Previous work has shown that this released phytol can be sequestered as phytol esters or used to synthesize tocopherols or phylloquinone. The phytyl group in phytyl diphosphate also can be recycled into chlorophyll by reaction with chlorophyllide *a* (not shown). Gutbrod *et al.* (1) use improved analytical methods to detect phytenal, suggesting that phytol can also be degraded in plants.

Gutbrod *et al.* (1) now present a strong case for the existence of this degradative pathway in plants. They first established a gas chromatography–MS method capable of detecting phytenal in nitrogen-deprived *Arabidopsis thaliana* leaves. Their experimental approach required identification of a suitable agent to derivatize and thus stabilize the aldehyde and consideration of multiple isomers within their spectra. They subsequently developed a more sensitive quantitative method for phytenal detection using liquid chromatography with a triple quadrupole mass spectrometer to increase measurement sensitivity. Using these methods, they demonstrated that phytenal levels increased when the plants were subjected to salt stress or dark, but not when subjected to heat or cold, which are stresses that do not produce chlorosis.

To demonstrate that the phytenal comes from phytol, they increased the levels of phytol in the plants without affecting the level of chlorophyll by placing the plants in liquid medium containing phytol. The added phytol resulted in accumulation of high levels of phytenal, with phytenal levels dropping when the plants were removed from the phytol-containing medium. The authors also tested a mutant blocked in pheophorbide *a* catabolism, in which chlorophyll hydrolysis is suppressed; phytenal did not accumulate in this mutant, providing additional evidence for a direct chemical relationship between chlorophyll breakdown and phytenal synthesis. Notably, the formation of phytenal occurred in boiled plants to about half the extent that it occurred in living plants, suggesting that the formation of phytenal may occur by a nonenzymatic mechanism, as well as *via* an enzymatic one. Finally, Gutbrod *et al.* (1) examined the relative proportions of phytenal and the known derivatives of phytol, finding that phytenal is a minor component of the overall pool, with the majority of the carbon being funneled into tocopherol and fatty acid phytyl ester synthesis. However, the authors also demonstrated that production of phytenal is enhanced when flux through either of these synthetic pathways is blocked by mutations, indicating that the three pathways compete for phytol substrate.

Questions remain about the reactions in the degradative pathway for phytol from chlorophyll metabolism, the identities of the enzymes that catalyze them, and their subcellular locations. It is also unclear whether this pathway serves to metabolize significant amounts of phytol from breakdown of phytyl esters, which decrease in amount when chlorotic stresses are relieved (7). The mechanism of the conversion of phytol to phytenal in particular remains ambiguous, as it seems to be at least partially nonenzymatic. Previous work had suggested a photooxidative reaction (10), but that is unlikely to be the only mechanism because Gutbrod *et al.* (1) show that phytenal can be produced during extended darkness. Because the degradative pathway for phytol has been described in mammals, orthologous plant enzymes might offer obvious candidates. However, as Gutbrod *et al.* (1) did not detect the downstream derivatives of phytenal found in the mammalian pathway, it is possible that the catabolism of phytenal in plants occurs *via* a novel pathway. On the other hand, such metabolites might be extremely transient and therefore difficult to detect. Knowing the identity of the enzymes involved also will help determine the subcellular location of phytol degradation. In mammals, the initial steps of the pathway to degrade phytanoyl–CoA (α-oxidation and first rounds of β-oxidation) take place in the peroxisome, followed by further breakdown of the chain in the mitochondrion.

A suggestion that a phytol degradative pathway may play an important role in the life of a plant comes from work showing that mutations in a gene leading to accumulation of phytanoyl–CoA result in the inability of plants to survive extended darkness as well as WT plants do (9). The gene of interest encodes a mitochondrial protein, electron-transfer flavoprotein:ubiquinone oxidoreductase, which seems to be important for catabolism of a variety of substrates, and may therefore offer an additional clue to establishing a full metabolic pathway. The new analytical tools established by Gutbrod *et al.* open the door to much needed research on this fascinating system. Given that phytol breakdown is key to the synthesis of antioxidant pigments and vitamins in certain fruits and seeds, understanding the multiple fates of phytol has the potential to increase our ability to ensure that crop plants are resilient to chlorotic stresses and able to produce nutritious food.

## Conflict of interest

The authors declare that they have no conflicts of interest with the contents of this article.

## References

[1] Gutbrod P., Yang W., Gruijicic G.V., Peisker H., Gutbrod K., Du L.F., Dörmann P. (2021). Phytol derived from chlorophyll hydrolysis in plants is metabolized via phytenal. J. Biol. Chem..

[2] Gutbrod K., Romer J., Dörmann P. (2019). Phytol metabolism in plants. Prog. Lipid. Res..

[3] Hörtensteiner S. (2006). Chlorophyll degradation during senescence. Annu. Rev. Plant Biol..

[4] Valentin H.E., Lincoln K., Moshiri F., Jensen P.K., Qi Q., Venkatesh T.V., Karunanandaa B., Baszis S.R., Norris S.R., Savidge B., Gruys K.J., Last R.L. (2006). The Arabidopsis *vitamin E pathway gene5-1* mutant reveals a critical role for phytol kinase in seed tocopherol biosynthesis. Plant Cell.

[5] vom Dorp K., Hölzl G., Plohmann C., Eisenhut M., Abraham M., Weber A.P.M., Hanson A.D., Dörmann P. (2015). Remobilization of phytol from chlorophyll degradation is essential for tocopherol synthesis and growth of Arabidopsis. Plant Cell.

[6] Shimada H., Ohno R., Shibata M., Ikegami I., Onai K., Ohto M.A., Takamiya K. (2005). Inactivation and deficiency of core proteins of photosystems I and II caused by genetical phylloquinone and plastoquinone deficiency but retained lamellar structure in a T-DNA mutant of Arabidopsis. Plant J..

[7] Lippold F., vom Dorp K., Abraham M., Hölzl G., Wewer V., Lindberg Yilmaz J., Lager I., Montandon C., Besagni C., Kessler F., Stymne S., Dörmann P. (2012). Fatty acid phytyl ester synthesis in chloroplasts of Arabidopsis. Plant Cell.

[8] van den Brink D.M., van Miert J.N., Dacremont G., Rontani J.F., Wanders R.J. (2005). Characterization of the final step in the conversion of phytol into phytanic acid. J. Biol. Chem..

[9] Ishizaki K., Larson T.R., Schauer N., Fernie A.S., Graham I.A., Leaver C.J. (2005). The critical role of *Arabidopsis* electron-transfer flavoprotein:ubiquinone oxidoreductuase during dark-induced starvation. Plant Cell.

[10] Rontani J.-F., Cuny P., Grossi V. (1996). Photodegradation of chlorophyll phytanol side chain in senescent leaves of higher plants. Phytochem.

